# Linguistic and Behavioral Mechanisms of Cyberbullying Among Chinese Social Media Users: A Scoping Review

**DOI:** 10.3390/bs16091627

**Published:** 2026-09-11

**Authors:** Shuangquan Deng, Azianura Hani Shaari, Azahah Abu Hassan Shaari, Yangfeng Gu

**Affiliations:** 1 Pusat Kajian Bahasa dan Linguistik/Center for Research in Language and Linguistics, Fakulti Sains Sosial dan Kemanusiaan/Faculty of Social Sciences and Humanities, Universiti Kebangsaan Malaysia/The National University of Malaysia, Bangi 43600, Malaysia; azianura@ukm.edu.my (A.H.S.); p122776@siswa.ukm.edu.my (Y.G.); 2 Pusat Kajian Psikologi dan Kesejahteraan Manusia/Centre for Research in Psychology and Human Well-Being, Fakulti Sains Sosial dan Kemanusiaan/Faculty of Social Sciences and Humanities, Universiti Kebangsaan Malaysia/The National University of Malaysia, Bangi 43600, Malaysia; azahah@ukm.edu.my

**Keywords:** cyberbullying, Chinese social media, linguistic behavior, pragmatic meaning, platform amplification

## Abstract

Cyberbullying among Chinese social media users has been described in relation to hostile words, pragmatic meanings, group alignment, identity positioning, and platform-mediated amplification. This scoping review mapped the linguistic and behavioral mechanisms reported in English-language studies indexed in Scopus and Web of Science concerning target-directed harm in Chinese social media contexts. Following the Arksey and O’Malley framework, Levac refinements, and PRISMA-ScR guidance, searches of Scopus and Web of Science identified 592 records. The 2015–2025 publication-date criterion was applied during screening. After deduplication, screening, and full-text eligibility assessment, 19 sources were included. Data were charted and synthesized thematically across lexical and lexico-semantic features, pragmatic strategies, discursive processes, and platform conditions. Four patterns emerged: evaluative labeling and semantic degradation; indirect aggression conveyed through irony, metaphor, insinuation, and culturally situated meanings; participant alignment and identity construction associated with legitimized hostility; and repetition, visibility, and collective participation associated with amplified harmful communication. Within this indexed English-language evidence base, cyberbullying was recurrently represented as a cumulative communicative process in which linguistic actions acquired harmful force through interpretation, social positioning, and social and technological circulation. Effective identification therefore requires attention to contextual meaning, interactional sequences, target relations, and platform dynamics, rather than reliance on isolated offensive terms. The review offers a provisional behavioral–linguistic framework derived from this indexed English-language evidence base while identifying methodological, platform, language, and database gaps for future empirical research.

## 1. Introduction

### 1.1. Conceptual Cyberbullying and Adjacent Forms of Online Harm

Social media has transformed interpersonal aggression by enabling harmful communication to be produced, circulated and reinforced across networked audiences. Cyberbullying is commonly defined as deliberate and repeated harm enacted through electronic communication, frequently involving an imbalance of power between perpetrators and targets (Kowalski et al., 2014). More recent syntheses, however, show that the defining attributes of cyberbullying remain contested: harm and digitally mediated aggression are broadly retained, but repetition, intentionality, anonymity and power imbalance are operationalized inconsistently across contemporary studies (Ray et al., 2024; Kim et al., 2026). In social media environments, however, repetition and power do not necessarily arise exclusively from recurring encounters between two individuals. Harm may develop through reposting, tagging, coordinated commentary, audience participation and the persistent visibility of content. A single message can therefore become part of a wider pattern of collective condemnation, reputational damage or identity-based attack.

From a behavioral-science perspective, cyberbullying can be understood as a set of communicative and social actions through which users target individuals, align with or resist other participants, and reinforce negative evaluations. Linguistic analyses of Chinese social media interactions show that lexical choices, address forms, evaluative constructions and pragmatic strategies can enact humiliation, stigmatization and negative identity positioning (Y. Xu, 2020; Zhong et al., 2022; S. Xu & Zhang, 2025). Such aggression may also develop cumulatively through repeated evaluations and participant uptake, allowing individually produced messages to contribute to broader networked patterns of blame or hostility (Y. Xu, 2020; Jin & Tay, 2023; Zhong et al., 2022). Examining language therefore provides insight not only into message content but also into the interactional processes through which aggressive behavior is enacted and socially coordinated.

The conceptual boundaries of cyberbullying nevertheless remain contested. Cyberbullying intersects with online harassment, hate speech, trolling, flaming and public shaming, but these concepts are not interchangeable. Online harassment may involve persistent unwanted communication without the repetition or power relations commonly associated with bullying. Hate speech primarily targets individuals or groups through protected or socially salient identities, whereas trolling is generally oriented towards provocation. Flaming refers to openly hostile exchanges that may not involve sustained targeting (Hardaker, 2010; Ortiz, 2020; S. Xu & Trzaskawka, 2021). Recent systematic reviews likewise report persistent inconsistency in how cyberbullying is distinguished from adjacent forms of online harm, reinforcing the need for explicit operational criteria in social media research (Ray et al., 2024; Kim et al., 2026).

Following established conceptualizations and recent syntheses, this review operationalizes cyberbullying as target-directed harmful communication involving repetition or persistence, including collectively distributed recurrence, together with an asymmetrical capacity to cause social, emotional or reputational harm (Kowalski et al., 2014; S. Xu & Trzaskawka, 2021; Ray et al., 2024; Kim et al., 2026). In networked environments, repetition may be produced not only by a single perpetrator but also through multiple users who reproduce the same label, accusation or negative stance, thereby generating cumulative targeting (Y. Xu, 2020; S. Xu & Trzaskawka, 2021). Accordingly, studies using adjacent terms such as online harassment, hate speech or toxic language were retained only when the communication examined fulfilled these operational conditions. This decision rule prevents generally offensive online communication from being treated automatically as cyberbullying.

### 1.2. Linguistic, Pragmatic and Platform-Related Mechanisms

Language can function as aggressive behavior at several interconnected levels. At the lexical level, swear words, derogatory labels, pronouns and emotionally charged expressions may explicitly position a target negatively. Ordinary words can also acquire harmful meanings when repeatedly associated with a particular person or identity. At the pragmatic level, aggression may be conveyed through sarcasm, presupposition, implicature, rhetorical questions and indirect speech act whose harmful force depends on contextual interpretation (Culpeper, 2011; Culpeper & Hardaker, 2017). At the discursive level, repeated labels, metaphors and evaluative descriptions may construct the target as morally deficient, socially undesirable or deserving of punishment (Van Dijk, 2005).

These mechanisms demonstrate that cyberbullying harm does not always reside in an isolated word or sentence. It may emerge cumulatively through the relationship between linguistic form, interactional context, participant alignment and audience uptake. An individual expression may appear ambiguous when examined independently but become recognizably aggressive when interpreted alongside preceding comments, repeated evaluations and patterns of collective response. This cumulative structure complicates the distinction between legitimate criticism, isolated offensive expression and sustained cyberbullying.

These context-dependent and adaptive forms also complicate automated moderation, because decontextualized lexical filters may miss indirect, coded or rapidly changing expressions (Davidson et al., 2017; Rosa et al., 2019). This detection challenge further underscores the need to analyze linguistic form together with pragmatic and interactional context. Recent surveys reinforce this limitation by identifying contextual information, linguistic diversity, dataset quality and the integration of socio-behavioral dynamics as continuing challenges for cyberbullying detection (Kiritchenko et al., 2021; Mishra et al., 2024; Lekscha & Mirbabaie, 2025; Philipo et al., 2026).

These interpretive challenges are particularly relevant to Chinese-language social media. Communication involving Chinese users is shaped by diverse linguistic varieties, cultural expectations and platform-specific regulatory environments. Previous research has highlighted the importance of face, relational positioning, indirect evaluation and moral judgment in Chinese interaction, although these practices should not be treated as uniform across all users or contexts (Gao et al., 1998; Spencer-Oatey, 2007; Yang et al., 2021). Chinese digital communication also involves rapidly evolving internet slang, homophonic substitutions, culturally specific metaphors and hybrid linguistic forms. Their meanings may vary across platforms and communities and may remain opaque to outsiders or automated systems.

Taken together, existing studies indicate that cyberbullying in Chinese social media environments is not reducible to a stable inventory of abusive words. Across linguistic, pragmatic and discourse-oriented research, explicit insults coexist with ordinary expressions whose harmful force emerges through recontextualization, repeated association, semantic prosody, metaphor and pragmatic implication (J. Li, 2020; J. Zhang, 2020; Y. Xu, 2020; Y. Li & Zhou, 2025; S. Xu & Zhang, 2025). At the same time, these linguistic resources can position targets as violators of moral, gendered, national or political expectations and can invite other participants to align with negative evaluations (Yuan & Liu, 2023; Zhong et al., 2022). The literature therefore converges on a relational view of online aggression in which lexical form, pragmatic interpretation, identity positioning and audience participation interact, although the relative contribution of these mechanisms varies across studies and contexts.

Platform conditions further complicate this relationship. Cross-platform evidence indicates that anonymity, publicity, community norms, content format and regulatory structures can influence how cyberbullying is produced and experienced across social media environments (Kennedy et al., 2025). In Chinese social media contexts, network analysis has shown how discourse can diffuse through the public communication structures of Sina Weibo around a highly visible event (Fang et al., 2024), while experimental evidence indicates that mandatory IP-location disclosure can suppress cyberbullying behavior across Douyin, Bilibili and Sina Weibo (Meng et al., 2026). Platform affordances are therefore treated in this review as contextual conditions shaping visibility, participation and amplification rather than as deterministic causes of cyberbullying.

### 1.3. Evidence Heterogeneity and Rationale for the Scoping Review

The heterogeneity of the evidence base provides a further rationale for this scoping review. Several included studies adopted case-based designs: Y. Xu (2020) and S. Xu and Zhang (2025) each examined an individual cyberbullying case on Sina Weibo, while Fang et al. (2024) analyzed discourse diffusion surrounding the 2022 Tangshan restaurant attack. By contrast, Zhong et al. (2022) analyzed 23,980 comments from five cases across multiple Chinese social media platforms, Chen et al. (2024) used a large computational dataset, and Meng et al. (2026) employed three experiments. Variation in unit of analysis, platform, method, terminology and operational definition therefore makes it difficult to determine which linguistic and behavioral mechanisms recur across contexts and which may be case-, platform- or method-specific. A scoping review is appropriate for mapping the breadth and characteristics of such heterogeneous evidence, clarifying conceptual boundaries and identifying research gaps rather than estimating a pooled effect (Arksey & O’Malley, 2005; Levac et al., 2010; Peters et al., 2020). Recent syntheses also identify two continuing gaps in the wider field: cyberbullying among adults remains comparatively underexplored, while technical detection research often insufficiently incorporates social, contextual and behavioral dynamics (Batool et al., 2025; Lekscha & Mirbabaie, 2025).

### 1.4. Review Aim and Research Questions

Accordingly, this scoping review maps and synthesizes research on the linguistic and behavioral mechanisms of cyberbullying involving Chinese users on social media. Rather than presenting previous studies as independent findings, the review compares evidence across lexical and lexico-semantic resources, pragmatic and discursive strategies, participant alignment and platform conditions in order to identify recurring patterns, contextual variation and gaps in the evidence base. Because several included publications originate from recurring author clusters, publication-level recurrence is distinguished from independent replication across unrelated research teams.

The review addresses four research questions: RQ1: What are the main characteristics, methodological approaches and social media contexts of indexed English-language research on cyberbullying involving Chinese users? RQ2: What recurrent linguistic and behavioral mechanisms of cyberbullying are identified across the included sources? RQ3: How do interactional context and platform affordances relate to the interpretation, reinforcement and amplification of cyberbullying in the reviewed literature? RQ4: What methodological, platform, language and evidence-base biases or gaps limit interpretation and transferability beyond the indexed English-language evidence base?

By answering these questions through comparative synthesis across the included publications, the review conceptualizes cyberbullying as a cumulative communicative behavior in which linguistic actions acquire harmful force through pragmatic interpretation, social positioning, participant alignment and platform-mediated circulation. This integrated framing distinguishes patterns reported across multiple publications from findings that remain platform-, case- or method-specific, while recognizing that recurrence across publications does not necessarily constitute independent replication when sources share author groups.

## 2. Materials and Methods

### 2.1. Review Design and Reporting Framework

This study employed a scoping review design to map and synthesize evidence on the linguistic and behavioral mechanisms of cyberbullying involving Chinese users on social media. The design was appropriate because terminology, theoretical framing, research design, analytical method, and platform coverage were heterogeneous; the aim was to characterize the evidence, clarify conceptual boundaries, and identify recurrent mechanisms and gaps rather than estimate pooled effects.

The review was conducted using the methodological framework developed by Arksey and O’Malley (2005) and subsequently refined by Levac et al. (2010). This framework informed the formulation of the review question, identification of relevant sources, study selection, data charting and synthesis of the evidence. The Population–Concept–Context framework was used to structure the review scope, eligibility criteria and search concepts.

**Figure 1:**
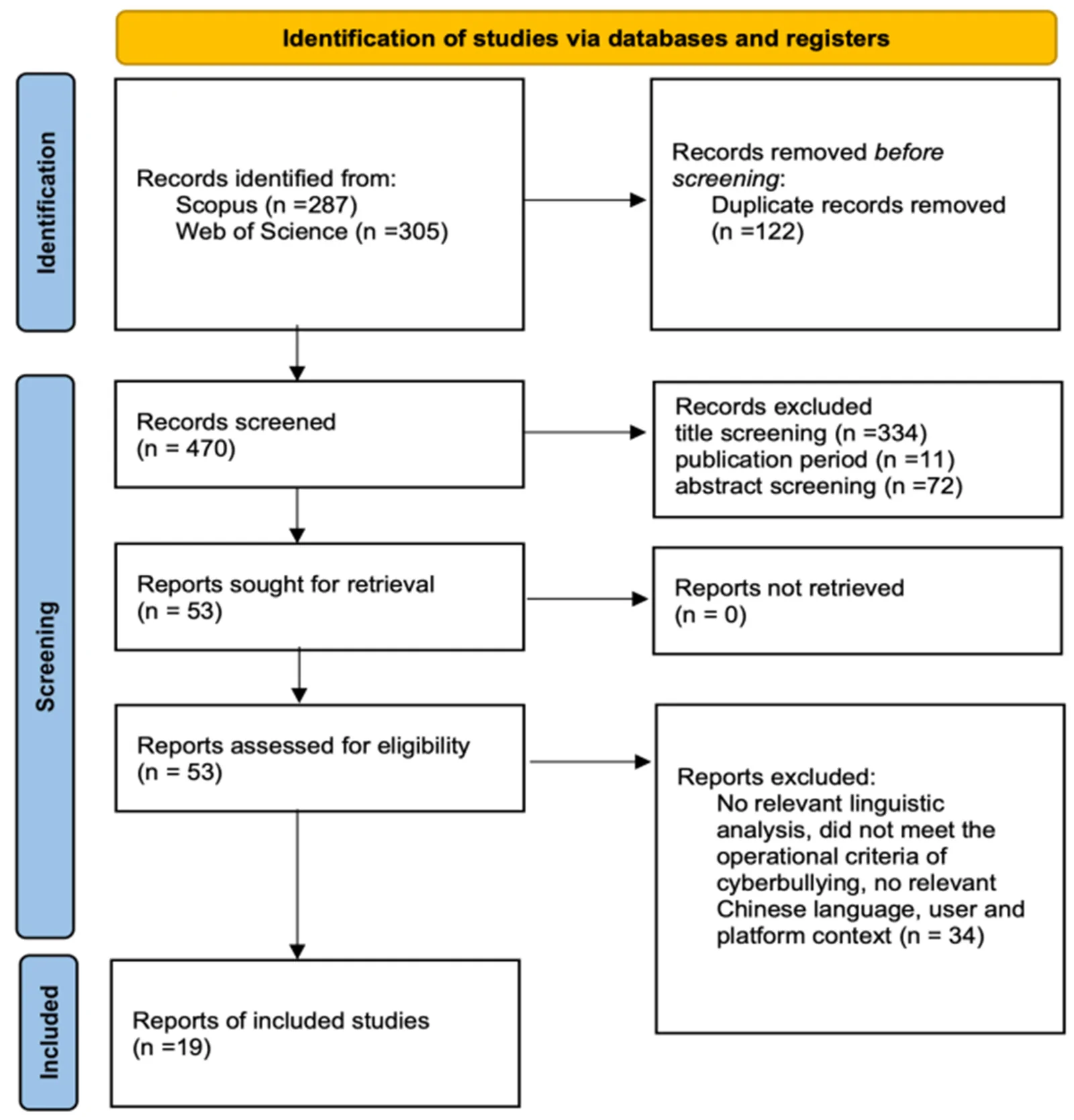
Flow diagram of source selection for the scoping review.

Reporting of this scoping review followed the Preferred Reporting Items for Systematic Reviews and Meta-Analyses Extension for Scoping Reviews (PRISMA-ScR; Tricco et al., 2018). A completed PRISMA-ScR checklist accompanies the revised submission, and the study-selection process, including identification, screening, eligibility assessment, and final inclusion of sources of evidence, is presented in the completed PRISMA flow diagram in Figure 1. Because no formal critical appraisal of individual sources was undertaken, the corresponding optional PRISMA-ScR critical-appraisal items are reported as not applicable.

The review protocol and related review materials were registered on Open Science Framework at https://osf.io/kf5s4 (accessed on 5 September 2026). To support transparency and reproducibility, the manuscript reports the eligibility criteria, complete database search strategies, screening procedures, data-charting categories, and synthesis approach.

### 2.2. Formulating the Research Question

The review question was formulated using the Population–Concept–Context framework, which is recommended for defining the scope, eligibility criteria and search concepts of scoping reviews (Peters et al., 2020). The population comprised users who produced, received or were represented in Chinese-language social media communication. This included Chinese-speaking users residing within and outside mainland China. Eligibility was determined primarily by the linguistic and communicative context examined rather than by assumptions concerning users’ nationality or geographical location.

The concept encompassed the linguistic and behavioral mechanisms through which cyberbullying was enacted, reinforced, interpreted or legitimized. Linguistic mechanisms included lexical and lexico-semantic choices, grammatical constructions, metaphors, pragmatic strategies and discursive processes through which online harm acquired contextual meaning (Culpeper, 2011; Y. Xu, 2020). Behavioral mechanisms included direct targeting, repeated negative evaluation, participant alignment, norm enforcement, moderation evasion and collective amplification. The context comprised interactions occurring on Chinese and international social media platforms, including Weibo, WeChat, Douyin or TikTok and other platforms containing relevant Chinese-language communication.

For the purposes of study selection, cyberbullying was operationally defined as target-directed online communication that caused, intended to cause or had the capacity to cause social, emotional or reputational harm. This definition was informed by established conceptualizations emphasizing intentional or consequential harm, repetition and power asymmetry (Kowalski et al., 2014; Tokunaga, 2010). Eligible communication was required to involve an identifiable individual or group, harmful linguistic or discursive conduct, repetition, persistence or collective amplification, and an asymmetrical capacity to harm or marginalize the target.

In social media environments, repetition could arise from one user or be distributed collectively through reposting, tagging, coordinated commentary, repeated labeling, or cumulative participation (Y. Xu, 2020; S. Xu & Trzaskawka, 2021). Studies using adjacent concepts such as online harassment, hate speech, toxic language, trolling, flaming, or public shaming were included only when these operational criteria were met; terminology or general offensiveness alone was insufficient (Hardaker, 2010; Ortiz, 2020; S. Xu & Trzaskawka, 2021).

The four research questions were mapped directly onto the charted evidence: study characteristics and methodological contexts addressed RQ1; linguistic, pragmatic, discursive, and behavioral findings addressed RQ2; interactional and platform conditions addressed RQ3; and evidence-base biases and gaps addressed RQ4.

### 2.3. Systematic Search Strategy

Scopus and Web of Science were selected deliberately to define a transparent and reproducible corpus of internationally indexed, peer-reviewed scholarship across linguistics, communication, behavioral sciences, social sciences and computational research. Their field-specific search functions, Boolean operators and filtering facilities support precise database-specific retrieval, while their interdisciplinary coverage is appropriate for mapping how Chinese cyberbullying has been represented across internationally visible research domains (Mohd Ali Piah et al., 2026). However, this database choice was not intended to provide exhaustive coverage of scholarship on Chinese-language cyberbullying. For a linguistically focused review, restricting the evidence base to internationally indexed sources carry a substantive validity cost because locally published Chinese-language research may contain more granular analyses of platform-specific slang, homophones, metaphors, pragmatic meanings and culturally situated interaction. The review therefore treats Scopus and Web of Science as defining a bounded indexed evidence slice rather than as representing the entire field.

The search strategy comprised three conceptual blocks representing online aggression, Chinese linguistic or platform contexts, and linguistic or discourse analysis. Broader terms such as online harassment, hate speech, toxic language, trolling, and flaming increased retrieval sensitivity, but records were retained only when they met the operational criteria for target-directed cyberbullying. The complete database-specific search strategies are presented in Table 1.

The database searches were conducted on 15 March 2026, with all queries and retrieval counts documented before deduplication. Publication-year and language criteria were applied during screening; eligible sources were English-language publications from 2015 to 2025. This restriction bounded the review to the prespecified Scopus/Web of Science evidence base and is treated as a substantive limitation rather than evidence of comprehensive coverage of Chinese-language scholarship. For online-first publications assigned to a later volume, the first online date determined eligibility; Meng et al. was first published online on 16 December 2025 and was eligible under the 2015–2025 window, although the final bibliographic record is assigned to Scientific Reports volume 16 (2026).

### 2.4. Study Selection

All records were exported to EndNote 2025 (Clarivate, Philadelphia, PA, USA) for reference management and deduplication using title, authorship, year, and DOI, followed by manual checking. Two reviewers then conducted title/abstract screening and full-text assessment; uncertain records progressed to full-text review, and disagreements were resolved by consensus.

During title and abstract screening, records were excluded when they clearly fell outside the review scope, including offline bullying, non-Chinese contexts, general social media behavior, psychological outcomes or prevalence without communicative analysis, and general sentiment/toxicity studies lacking a link to target-directed cyberbullying.

Full-text publications were eligible when they reported original empirical research examining cyberbullying or substantively equivalent target-directed online aggression involving Chinese-language users or Chinese-language communication on social media. Eligible studies were also required to provide meaningful and interpretable analysis of at least one relevant linguistic or behavioral dimension. These dimensions included lexical choices, lexico-semantic patterns, metaphors, grammatical constructions, pragmatic strategies, interactional sequences, participant alignment, discursive identity construction or platform-mediated amplification. Eligible publication types comprised peer-reviewed journal articles, full-length conference papers and scholarly book chapters reporting original empirical research. Dissertations, theses, book reviews, editorials, conference abstracts, non-research commentaries and publications without sufficient methodological information were excluded.

Studies focused only on prevalence, psychological outcomes, demographics, software architecture, model optimization, or classification performance were excluded unless they provided interpretable linguistic, contextual, or interactional findings. Studies using adjacent terminology were retained only when the operational criteria established above were satisfied.

### 2.5. Data Charting and Analysis

Data from the included sources were charted using a standardized extraction form developed according to the review question and the Population–Concept–Context framework. The extracted information comprised authorship, publication year, publication type, geographical and linguistic context, social media platform, dataset or sample, analytical method, theoretical framework and terminology used to describe the harmful behavior.

Findings were charted across lexical/lexico-semantic, pragmatic, discursive, behavioral, and platform-related domains. The extraction form also recorded whether and how each source established an identifiable target, harmful communicative conduct, repetition or collective amplification, and asymmetrical capacity to harm, allowing cyberbullying evidence to be distinguished from broader offensive-, abusive-, or toxic-language data.

For computational studies, only interpretable findings concerning linguistic, contextual, sequential or interactional features were incorporated into the principal synthesis. Classification accuracy, F1 scores and comparisons among computational models were treated as supplementary evidence and were not interpreted as direct evidence of linguistic or behavioral mechanisms.

No formal methodological quality, critical appraisal or study-level risk-of-bias assessment was undertaken because the review aimed to map the evidence rather than estimate an aggregate effect. Study design, dataset size, platform coverage, analytical method, and data-collection transparency were nevertheless considered when interpreting findings; the absence of formal appraisal is acknowledged as a limitation and reflected in the PRISMA-ScR checklist. Analysis proceeded in two stages. Descriptive mapping was first used to summarize publication years, publication types, platforms, data sources, methodological approaches and analytical orientations. Frequencies were reported only for characteristics that were sufficiently comparable across sources and were not interpreted as statistically generalizable estimates.

Analysis proceeded in two stages. Descriptive mapping summarized publication characteristics, platforms, data sources, methods, and analytical orientations. Thematic synthesis then compared findings across lexical/lexico-semantic realization, pragmatic meaning, discursive identity construction, behavioral reinforcement, and platform affordances to identify recurrent patterns, contextual variation, and divergent evidence.

The synthesis focused on recurring cross-source patterns rather than sequential study summaries. Author concentration was considered explicitly: recurrence across publications linked to the same author cluster was not treated as independent replication. The resulting framework therefore represents patterns within the indexed English-language evidence base rather than an exhaustive account of Chinese-language cyberbullying scholarship.

## 3. Results

Following study selection, the findings are reported in direct alignment with the four research questions stated in the Introduction: study characteristics and social media contexts (RQ1), recurrent linguistic and behavioral mechanisms (RQ2), interactional and platform conditions (RQ3), and evidence-base biases and research gaps (RQ4).

### 3.1. Study Selection

The searches identified 592 records (Scopus, *n* = 287; Web of Science, *n* = 305). After removal of 122 duplicates, 470 records remained; 334 were excluded at title screening and 11 fell outside the 2015–2025 publication window, leaving 125 records for abstract screening.

A further 72 records were excluded after abstract assessment. All 53 remaining full texts were retrieved and assessed; 34 were excluded for failing the linguistic/behavioral, cyberbullying, Chinese-context, publication-type, or methodological criteria.

Consequently, 19 sources of evidence met the eligibility criteria and were included in the final descriptive mapping and thematic synthesis. The complete study process is presented in Figure 1.

### 3.2. RQ1: Characteristics, Methodological Approaches and Social Media Contexts

The 19 included sources comprised 16 peer-reviewed journal articles and three full-length conference papers; no book chapters were included. They were first published online between 2019 and 2025, with Meng et al. (2026) eligible on the basis of its 16 December 2025 online-first date.

The sources represented diverse disciplinary and analytical traditions, including linguistics, pragmatics, discourse studies, social and behavioral research and computational modeling. Their methodological approaches included corpus analysis, content analysis, critical discourse analysis, pragmatic analysis, sentiment analysis, semantic-network analysis, questionnaire research, topic modeling and machine-learning classification. These categories were not mutually exclusive because several sources combined qualitative, quantitative and computational techniques. Explicit theoretical framing was uneven but included Bucholtz and Hall’s identity framework in S. Xu and Zhang (2025), Language Management Theory in J. Li (2019b), Discursive Psychology in Jin and Tay (2023), and Critical Discourse Analysis in Liu et al. (2021). Many other sources were primarily method-led rather than explicitly theory-led, indicating theoretical as well as methodological diversity within the evidence base.

Dataset size and unit of analysis varied from case-based linguistic samples to large computational/network datasets, questionnaires, and experiments. Sina Weibo was the most frequently represented platform (12/19), while the remaining studies covered messaging, short-video, forum, cross-national, and multiplatform environments. Detailed platform, dataset, and methodological characteristics are provided in Table 2 and Supplementary Table S1.

### 3.3. RQ2: Recurrent Linguistic and Behavioral Mechanisms

To answer RQ2, findings were compared across sources rather than restated study by study. Three recurrent mechanism clusters were identified: context-dependent linguistic aggression; indirect aggression, plausible deniability and cumulative harm; and identity regulation and collective moral positioning. These clusters are analytically related and together describe how lexical, pragmatic, interactional and identity-positioning resources can enact or reinforce target-directed harm.

#### 3.3.1. Context-Dependent Linguistic Aggression

Explicit and context-dependent aggression coexisted. Direct indicators included swear words, sexualized expressions, derogatory labels, pronouns, and negatively charged vocabulary (J. Li, 2019a, 2020; Zhong et al., 2022; J. Lu et al., 2023), while ordinary words such as “fairy” and “Gangjing” acquired derogatory force through target-specific recontextualization (S. Xu & Zhang, 2025; Xia & Wang, 2022). Homophones, misspellings, slang, symbols, and other variants also supported moderation evasion (J. Li, 2019a, 2019b), showing that lexical form alone was insufficient without context, cultural knowledge, and target identity.

#### 3.3.2. Indirect Aggression, Plausible Deniability and Cumulative Harm

Indirect aggression was frequently conveyed through implicature, presupposition, sarcasm, rhetorical questions, negation, indirect address, and semantic prosody, allowing harmful evaluations to retain plausible deniability (Y. Xu, 2020; J. Zhang, 2020; Zhong et al., 2022). Contextual and sequential information was central: repeated semantic associations, aligned responses, and participant-distributed repetition could transform individually ambiguous comments into cumulative targeting, extending the unit of analysis from isolated messages to communicative events (Chen et al., 2024; Y. Zhang et al., 2024).

#### 3.3.3. Identity Regulation and Collective Moral Positioning

Identity construction connected interpersonal attack with wider moral and social positioning. Evaluative labels and social categories portrayed targets as violating accepted values, while hostility could be framed as blame, justice, public accountability, or defense of collective norms (S. Xu & Zhang, 2025; Jin & Tay, 2023; Liu et al., 2021). Audience alignment was not universal: supportive and resistant responses were also observed (S. Lu et al., 2022), indicating a contingent rather than deterministic process.

### 3.4. RQ3: Interactional Context, Platform Affordances and Amplification

To answer RQ3, the review examined how surrounding discourse, participant responses, and platform conditions were associated with the interpretation and circulation of harmful communication. Across pragmatic and discourse-oriented studies, ambiguous expressions were interpreted as more clearly aggressive when preceding comments, repeated evaluations, and audience alignment were taken into account (Y. Xu, 2020; J. Zhang, 2020; Zhong et al., 2022). Interactional context was therefore repeatedly reported as relevant to the recognition of indirect harm and patterns of cumulative targeting, while platform conditions were examined in relation to the visibility, persistence, and circulation of these interactions.

Platform conditions were associated with visibility, persistence, participation, and circulation rather than treated as general causal effects. Weibo studies documented networked visibility and diffusion (Fang et al., 2024; S. Lu et al., 2022); WeChat evidence emphasized the accumulation of negative framing across longer discourse (J. Zhang, 2020); and experimental evidence linked mandatory IP-location disclosure with reduced cyberbullying participation under tested conditions (Meng et al., 2026).

**Figure 2:**
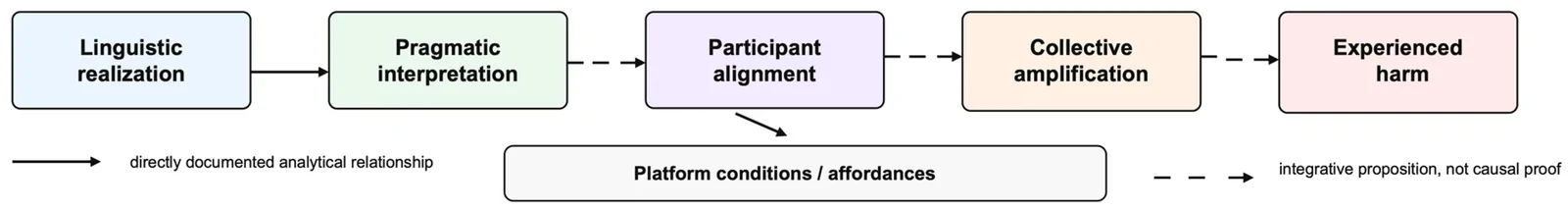
Provisional integrative analytical framework derived from the included indexed English-language evidence. Note. Figure 2 synthesizes relationships identified within the 19 English-language studies indexed in Scopus and Web of Science. It is not a tested causal model. Recurrently documented links concerned linguistic realization and contextual/pragmatic interpretation, and platform conditions and discourse visibility/circulation. The longer sequence from pragmatic interpretation to participant alignment, collective amplification, and experienced harm remains an integrative proposition; experienced harm was rarely measured directly. Recurrence within overlapping author clusters is treated as thematic consistency rather than independent replication, and the framework remains provisional pending broader Chinese-language and cross-platform testing.

Multiplatform and computational studies likewise indicated that domain context mattered for interpretation or detection (Zhong et al., 2022; Y. Zhang et al., 2024), while network analysis documented discourse diffusion through platform structures (Fang et al., 2024). However, direct cross-platform comparisons remained scarce, so the relationships summarized in Figure 2 are treated as provisional analytical associations rather than stable platform-specific behavioral effects.

Across RQ2 and RQ3, Figure 2 summarizes linked relationships among linguistic realization, pragmatic interpretation, participant alignment/identity positioning, and platform-mediated amplification. Because some publications share recurring author clusters, such recurrence is treated as thematic consistency rather than as independent replication or a tested temporal or causal pathway.

Table 3 provides an integrated mapping of the linguistic, behavioral and platform-related mechanisms identified in RQ2 and RQ3, together with representative sources.

### 3.5. RQ4: Evidence-Base Biases and Research Gaps

Five constraints were evident. First, Sina Weibo dominated platform coverage (12/19), with limited direct comparison across messaging, short-video, and forum settings. Second, study designs and units of analysis were highly heterogeneous. Third, victims’ interpretations, repetition/power asymmetry indicators, and experienced harm were often inferred rather than directly measured. Fourth, restriction to English-language Scopus/Web of Science publications may underrepresent Chinese-language scholarship. Fifth, six of the 19 sources were linked to recurring Xu- or Li-related author clusters, so publication-level recurrence cannot be treated as independent replication. These constraints bound the findings to the internationally indexed English-language evidence captured by the review.

#### Author and Analytical Diversity

Author concentration coexisted with analytical diversity across lexical/network analysis, Discursive Psychology, Critical Discourse Analysis, Conversation Analysis/pragmatics, computational modeling, network propagation, and experiments. Patterns recurring across distinct author groups and methods were therefore treated as stronger evidence of thematic consistency than patterns concentrated within one author cluster.

Accordingly, the RQ findings should not be generalized to Chinese-language scholarship as a whole; the broader implications of these evidence-base limitations are considered in Section 4.

## 4. Discussion

### 4.1. Behavioral Interpretation of the Findings

This scoping review indicates that cyberbullying involving Chinese users can be analyzed as a multilevel communicative process rather than as a collection of isolated offensive terms. Across the included publications, lexical choices, pragmatic inference, identity positioning, participant alignment and platform circulation were repeatedly reported in relation to one another. However, because several publications originate from recurring author clusters, this recurrence is interpreted as thematic consistency within the reviewed evidence rather than as independent replication. The contribution is therefore integrative rather than prevalence-based: the heterogeneous evidence identifies analytical links among linguistic realization, social interpretation, learned adaptation, collective behavior and norm enforcement, but does not establish a single causal sequence or estimate how frequently any mechanism occurs.

The first major finding is that lexical form alone is insufficient for identifying cyberbullying. Explicit insults and derogatory labels provide visible indicators of aggression, but ordinary expressions, homophones, misspellings and culturally situated labels can acquire harmful force through recontextualization and repeated association with a target (J. Li, 2019a, 2019b, 2020; Y. Xu, 2020; J. Zhang, 2020; Zhong et al., 2022; S. Xu & Zhang, 2025). This helps explain why apparently neutral expressions may become aggressive only when their local discourse history, target identity and shared cultural knowledge are considered. The analytical implication is that the relevant unit is not simply the individual word or message but the interactional sequence in which meaning is negotiated and accumulated.

A second major finding concerns indirect aggression. Implicature, presupposition, sarcasm, rhetorical questions, metaphor and semantic prosody allow negative evaluations to be communicated without an explicit abusive proposition (Y. Xu, 2020; J. Zhang, 2020; Zhong et al., 2022). Such forms can create plausible deniability because the harmful interpretation depends partly on contextual inference rather than literal wording. This feature is especially important for both human interpretation and automated detection: a decontextualized message may appear ambiguous, whereas the same message becomes recognizably hostile when preceding comments, repeated evaluations and participant responses are considered. The reviewed evidence therefore supports treating pragmatic inference as a central analytical bridge between linguistic realization and perceived aggression.

A third finding is that repetition and power can be socially distributed across participants rather than produced only through repeated acts by one perpetrator. Several studies reported multiple users reproducing the same accusation, label or evaluative stance, thereby creating cumulative targeting even when individual contributors participated only once (Y. Xu, 2020; S. Xu & Trzaskawka, 2021; Jin & Tay, 2023; Liu et al., 2021; S. Xu & Zhang, 2025). Identity-based framing further positioned targets against moral, gendered, national or social expectations, allowing hostility to be presented as correction, accountability or defense of collective values. This broadens the conventional dyadic understanding of repetition and power by showing how audience alignment and norm enforcement can distribute aggressive participation across a network. At the same time, supportive or resistant responses reported by S. Lu et al. (2022) indicate that audience alignment is contingent rather than automatic.

The fourth finding concerns platform conditions. The reviewed studies associated platform structures with differences in visibility, persistence, participation and circulation, but the evidence does not support treating these affordances as deterministic causes of cyberbullying (Fang et al., 2024; J. Zhang, 2020; Meng et al., 2026). Rather, platform conditions shape the communicative environment in which linguistic and interactional processes become more or less visible, repeatable or collectively accessible. Figure 2 therefore integrates linguistic realization, pragmatic interpretation, participant alignment and identity positioning, and platform-mediated amplification as a provisional set of analytical relationships. Relationships between linguistic form and contextual interpretation, and between platform conditions and visibility or circulation, were more directly documented, whereas the longer pathway toward experienced harm remains an integrative proposition requiring longitudinal and mixed-method testing.

### 4.2. Implications for Detection, Governance and Practice

The findings support context-sensitive detection and moderation rather than reliance on profanity lists or isolated-message classification. Systems should account for lexical variation, homophones, character substitutions, culturally situated expressions and surrounding interactional context, with automated outputs treated as risk indicators subject to human review in ambiguous cases (J. Li, 2019a, 2019b; Chen et al., 2024; Y. Zhang et al., 2024).

For governance and education, the main implication is to distinguish isolated offense from sustained target-directed harm by considering identifiable targeting, cumulative repetition or amplification, identity-based degradation and asymmetrical capacity to harm (S. Xu & Trzaskawka, 2021). Digital-literacy interventions can likewise address how participant alignment may reinforce aggression or, conversely, how resistance and supportive responses may interrupt it (S. Lu et al., 2022).

### 4.3. Limitations, Biases and Future Research

The findings should be interpreted within a bounded and heterogeneous evidence base. Only 19 English-language sources indexed in Scopus and Web of Science were included; Sina Weibo was disproportionately represented, methods and units of analysis were non-equivalent, and six sources (31.6%) came from two recurring author clusters. No formal methodological quality or study-level risk-of-bias appraisal was undertaken, consistent with the mapping purpose of the review. These conditions mean that thematic recurrence should not be interpreted as prevalence, effect size or independent replication, and the indexed corpus may underrepresent locally published Chinese-language concepts and platform-specific practices.

Victims’ interpretations, power asymmetry and experienced harm were also often inferred from textual patterns, public visibility or collective participation rather than measured directly. Future research should therefore combine Chinese-language and bilingual evidence with broader cross-platform, longitudinal and mixed-method designs that connect discourse patterns with audience responses and targets’ reported experiences. Such work would allow the provisional relationships summarized in Figure 2 to be tested more directly and would clarify their transferability beyond the current indexed evidence base.

## 5. Conclusions

This scoping review synthesized 19 English-language studies indexed in Scopus and Web of Science on the linguistic and behavioral mechanisms of cyberbullying involving Chinese social media users. The evidence suggests that cyberbullying is better understood as a context-dependent and socially distributed communicative process than as the use of isolated offensive words. Across the reviewed literature, lexical and pragmatic resources, identity positioning, participant alignment and platform-mediated circulation were recurrently associated with cumulative targeting. The review therefore proposes a provisional, non-causal analytical framework linking linguistic realization, pragmatic interpretation, participant alignment and collective amplification. However, the evidence base remains limited by platform concentration, English-language database coverage and the limited direct measurement of victim harm. Future research should test these relationships using Chinese-language evidence, broader cross-platform comparisons and mixed-method or longitudinal designs.

## Figures and Tables

**Table 1:** Database-specific search strategies used in Scopus and Web of Science. Note. The asterisk (*) denotes truncation and was used to retrieve multiple lexical variants sharing the same word stem (e.g., cyberbully, cyberbullying, cyberbullied).

Database	Complete Search String
Scopus	TITLE-ABS-KEY ((cyberbully* OR “online aggression” OR “online harassment” OR “digital bullying” OR “internet bullying” OR “abusive language” OR “toxic language” OR “hate speech” OR “online hate speech” OR trolling OR flaming OR “public shaming”) AND (Chinese OR China OR Mandarin OR Cantonese OR “Chinese language” OR “Chinese-language” OR Weibo OR WeChat OR Douyin OR TikTok OR Zhihu OR Bilibili) AND (language OR linguist* OR lexical* OR semantic* OR pragmatic* OR discourse OR metaphor* OR impoliteness OR “speech act*” OR communication OR interaction))
Web of Science	TS = ((cyberbully* OR “online aggression” OR “online harassment” OR “digital bullying” OR “internet bullying” OR “abusive language” OR “toxic language” OR “hate speech” OR “online hate speech” OR trolling OR flaming OR “public shaming”) AND (Chinese OR China OR Mandarin OR Cantonese OR “Chinese language” OR “Chinese-language” OR Weibo OR WeChat OR Douyin OR TikTok OR Zhihu OR Bilibili) AND (language OR linguist* OR lexical* OR semantic* OR pragmatic* OR discourse OR metaphor* OR impoliteness OR “speech act*” OR communication OR interaction))

**Table 2:** Characteristics and principal findings of the included sources of evidence (*n* = 19). (*) denotes truncation and was used to retrieve multiple lexical variants sharing the same word stem.

Source	Platform(s)	Dataset or Sample	Analytical Approach	Primary Analytical Focus
**Y. Xu (2020)**	Sina Weibo	Thousands of comments from one case; exact *n* not reported	Corpus and pragmatic analysis	Indirect and cumulative aggression
**S. Xu and Zhang (2025)**	Sina Weibo	Comments from one case; exact *n* not reported	Corpus-based identity analysis	Victim identity construction
**J. Li (2019a)**	Sina Weibo	Selected language samples; exact *n* not reported	Content analysis	Offensive and context-dependent vocabulary
**J. Li (2019b)**	Sina Weibo	Selected offensive-language samples; exact *n* not reported	Inductive content analysis	Linguistic variation and moderation evasion
**J. Li (2020)**	Chinese websites; mainly Weibo	Selected discourse samples; exact *n* not reported	Semantic, pragmatic and content analysis	Context-dependent bullying language
**S. Xu and Trzaskawka (2021)**	Cross-national cases, including China	Selected authentic cases	Interdisciplinary case analysis	Definitions, repetition and harm
**Y. Li and Peng (2024)**	Chinese social media	Questionnaire, *n* = 705, and textual examples	Content and questionnaire analysis	Textual and sentimental cues
**Zhong et al. (2022)**	Weibo, WeChat, QQ, TikTok and Toutiao	23,980 comments from five cases	Lexical, sentiment and network analysis	Explicit and implicit aggression
**S. Lu et al. (2022)**	Sina Weibo	53,526 posts	Sentiment analysis and LDA topic modeling	Emotional and public responses
**J. Zhang (2020)**	WeChat	26 texts; 18,722 tokens and 4266-word types	Corpus and pragmatic analysis	Semantic prosody and incitement
**Chen et al. (2024)**	Chinese social media and Douban	Expanded COLDATASET*: 38,991 texts total (28,991 training; 5000 validation; 5000 test). The final total includes approximately 1660 offensive remarks from ten cyberbullying incidents added during expansion, together with Douban one-star reviews	XLNet–Deep Bi-LSTM modeling	Context-sensitive detection
**Chao et al. (2024)**	PTT (Taiwan)	16,967 comments linked to pandemic events	Two-stage ML modeling, ChatGPT rephrasing, and LDA	Detecting and rephrasing pandemic hate speech to harmony
**Jin and Tay (2023)**	Sina Weibo	14,540 comments (HBRCF account)	Discursive Psychology (DP) and rhetorical analysis	Hateful comments as networked practice of blame/justice
**Liu et al. (2021)**	Sina Weibo	102,560 postso	Critical Discourse Analysis (CDA) and spatial analysis	Anti-African sentiments, racial stigma, and disease misinformation
**J. Lu et al. (2023)**	Zhihu and Tieba	12,011 comments (ToxiCN dataset)	Hierarchical taxonomy (Monitor Toxic Frame)	Fine-grained detection of toxic language (direct and indirect)
**Xia and Wang (2022)**	Baidu Post Bar	40 interactional threads from 6534 results	Conversation Analysis (CA)-informed approach	Communicative actions and responses to trolling label “Gangjing”
**Y. Zhang et al. (2024)**	Weibo, Zhihu, WeChat, Xiaohongshu, and video sites	19,089 texts (MCIHD dataset)	Domain-Enhanced Prompt Learning (DePL)	Multi-domain detection of implicit vs. explicit hate speech
**Fang et al. (2024)**	Sina Weibo	366,602 network communication nodes	Complex network modeling and degree analysis	Diffusion of feminist discourses (Tangshan attack case)
**Meng et al. (2026)**	Douyin, Bilibili, and Sina Weibo	1270 participants across three experiments	Scenario-based experimental design and regression	Mandatory IP disclosure impact on suppressing cyberbullying

Note. CDA = critical discourse analysis; XLNet = a context-sensitive pre-trained language model; Bi-LSTM = bidirectional long short-term memory; DePL = Domain-Enhanced Prompt Learning; LDA = latent Dirichlet allocation. Platform categories and analytical approaches were not mutually exclusive.

**Table 3:** Integrated mapping of linguistic, behavioral and platform-related mechanisms relevant to RQ2 and RQ3.

Analytical Domain	Synthesized Pattern	Function in Cyberbullying	Representative Sources
Lexical	Context-dependent derogatory labeling	Assigns negative identities through ordinary or evaluative words that become toxic in context (e.g., “fairy” to attack women or “Gangjing” for trollers).	J. Lu et al. (2023); Xia and Wang (2022); S. Xu and Zhang (2025); J. Li (2020)
Lexical	Linguistic evasion	Circumvents keyword moderation through homophones, character deformation, misspellings, and special symbols.	J. Lu et al. (2023); J. Li (2019a, 2019b)
Pragmatic	Implicature and presupposition	Communicates aggression indirectly (e.g., “cold language”), allowing for plausible deniability while maintaining hostility.	Y. Zhang et al. (2024); Jin and Tay (2023); Y. Xu (2020); Zhong et al. (2022)
Pragmatic	Sarcasm and ironic metaphors	Expresses or indexes harmful evaluation through humor or irony (e.g., animal metaphors such as “green cockroach”) without explicit insults.	J. Lu et al. (2023); Xia and Wang (2022); Chao et al. (2024); J. Zhang (2020); Zhong et al. (2022)
Interactional	Cumulative and networked aggression	Intensifies harm through repetition, participant alignment, and “networked practices of blame” that escalate hostility.	Jin and Tay (2023); Xia and Wang (2022); Fang et al. (2024); Y. Xu (2020); J. Zhang (2020)
Discursive	Identity construction and othering	Positions targets as socially, morally, or biologically deviant (e.g., racist nodal points or “Scarlet” institutional identities).	Liu et al. (2021); Jin and Tay (2023); S. Xu and Zhang (2025);
Platform-related	Visibility, amplification, and identity cues	Is associated with discourse visibility and persistence; experimental evidence also indicates reduced cyberbullying participation under identity-disclosure conditions.	Fang et al. (2024); Meng et al. (2026);
Technological	Context- and domain-sensitive detection	Captures implicit aggression and domain-specific rhetoric not detectable through isolated keywords.	Chao et al. (2024); Y. Zhang et al. (2024); J. Lu et al. (2023); Chen et al. (2024)

## Data Availability

The supporting data and materials for this scoping review are publicly available in Zenodo at https://doi.org/10.5281/zenodo.22348976. Additional review materials, including the review protocol and PRISMA-ScR reporting documentation, are available through the Open Science Framework at https://osf.io/kf5s4 (accessed on 5 September 2026).

## Supplementary Materials

The following supporting information can be downloaded at: https://www.mdpi.com/article/10.3390/bs16091627/s1, File S1 (Table S1): Source-level linguistic, behavioral and platform-related findings; File S2: Completed PRISMA-ScR checklist; File S3: PRISMA flow diagram for source identification, screening, eligibility and inclusion; File S4: Updated scoping review protocol.
