# Supplementary material for: Linguistic and Behavioral Mechanisms of Cyberbullying Among Chinese Social Media Users: A Scoping Review
Source: Behav Sci (Basel). 2026 Sep 11;16(9):1627. doi: 10.3390/bs16091627 (PMC13603809; doi:10.3390/bs16091627)
Supplement: Supplementary file 1 [file behavsci-16-01627-s001.zip › File S1.Supplementary_Table_S1.pdf]

Supplementary Table S1. Source-level linguistic, behavioral and platform-related findings.

Source	Publication type	Platform and context	Dataset or sample	Analytical approach	Principal linguistic or discursive findings	Behavioral or platform-related findings	Adjacent terminology / construct	Evidence of identifiable target	Evidence of repetition or collective amplification and inclusion rationale
Xu (2020)	Journal article	Sina Weibo; high-profile cyberbullying and defamation case	Thousands of targeted comments associated with one case; exact sample size not reported	Corpus linguistic and pragmatic analysis	Second-person pronouns, interrogative constructions, exclamations, address forms and evaluative grammatical resources were used to judge and negatively position the target. Harm emerged cumulatively through repeated interactions,	Weibo's public interaction enabled group targeting, repeated negative evaluation and collective trial by social media. Repetition was distributed across participating users rather than limited to one perpetrator.	Cyberbullying, cyber harassment, online bullying, verbal abuse, reputation infringement, defamation	Individual target: Yang Meiqing (mother of the deceased child Wang Fengya) and her family	Evidence: The study interprets harm as cumulative across repeated interactions distributed among participating Weibo users, described metaphorically as an 'invisible aggressive fist'. Rationale: Fits the review's

					producing an "invisible aggressive fist".				operational definition because it involves a specific individual target and distributed repetition across multiple users. The evidence supports analyst-inferred communicative harm; victim-reported outcomes were not directly measured in the linguistic analysis.
Xu and Zhang (2025)	Journal article	Sina Weibo; a Chinese	Manually compiled	Corpus-based identity analysis	Address forms and labels such as "fairy", "party	Repeated identity labelling encouraged	Cyberbullying, other-constructed victim identity,	Individual target: A female Sichuan	Evidence: Repeated identity labeling and

	cyberbullying case	comments associated with one incident; exact sample size not reported	informed by Bucholtz and Hall's identity framework	member" and "good student" constructed stigmatized victim identities. Implicature, stance-taking and relational positioning extended attacks from behaviour to gender, appearance and social background.	participant alignment and collectively positioned the target as morally untrustworthy. Public visibility expanded the attack beyond the original behaviour under discussion.	online misogyny, 'fairy' (negative label), cyber manhunt	University student (Z_W) who falsely accused a metro passenger	doxxing (manhunt) by numerous Weibo users expanded and prolonged the target-directed attack. The source reports an offline consequence, including termination of an internship, but the causal contribution of the online comments was not independently measured. Rationale: Fits because it documents public, collective and persistent negative identity construction

									directed at an identifiable individual.
Li (2019a)	Full-length conference paper	Sina Weibo	Selected cyberbullying-language samples; exact sample size not reported	Content analysis	Reported lexical categories included abusive expressions, sexualized expressions, curses, emotional or emphatic expressions, emerging vocabulary and ordinary or idiomatic words with specialized meanings. Sarcasm and contextual reinterpretation also conveyed aggression.	The coexistence of direct and context-dependent aggression demonstrated that user behaviour could not be identified reliably through profanity lists alone.	Cyberbullying, online language bullying behavior, offensive/sensitive language, flaming, online harassment	Individual targets: Case-specific individuals in public high-participation incidents on Weibo (e.g., Liu Xin/roommate)	Evidence: Harassment on Weibo was one-sided, longer-lived, and continuous, involving multiple rounds of sending offensive messages on comment walls or private letters. Rationale: Fits the definition as it conceptualizes cyberbullying as repeated, continuous, and offensive language-based aggression.

Li (2019b)	Full-length conference paper	Sina Weibo	Selected offensive-language samples; exact sample size not reported	Inductive content analysis informed by Language Management Theory	Linguistic features ranged from inherently offensive swear and taboo words to mild expressions that became offensive within a specific context. Tags, mentions, analogies and metaphors contributed to repetition and implicit aggression.	The study proposed graded detection combining lexical resources with contextual review. Tagging and mentions supported repeated targeting and wider audience participation.	Cyberbullying, offensive language/information, sensitive cyberbullying, trolling, deindividuation	Individual targets: Specific individuals tagged/mentioned in high-profile cases (e.g., Liu Xin)	Evidence: Platform features such as the '@' symbol (tagging/mentions) and hashtags were described as supporting repeated targeting and wider audience participation. Rationale: Fits the operational definition because the study links identifiable targeting with repeated, platform-enabled participation; harm or humiliation is interpreted at

									the communicative level rather than measured as a victim-reported outcome.
Li (2020)	Journal article	Chinese websites, with emphasis is on Sina Weibo	Selected examples of cyberbullying discourse; exact sample size not reported	Semantic, pragmatic and content analysis	Abusive, sexualized and strongly evaluative expressions were prominent. Ordinary expressions could also become offensive through contextual reinterpretation.	The findings indicated that aggressive communicative behaviour depended on the relationship among linguistic form, participant intention and interactional context. Lexical strength alone was insufficient for identifying harm.	Cyberbullying, cyber aggression, hate speech, flaming, online harassment	Individual or group targets of contentious debates on social platforms (e.g., celebrities, minors, and case targets)	Evidence: The study notes that indirect cyberbullying can be shared repeatedly across social platforms, increasing the reach and persistence of hostile communication. Rationale: Fits because it examines repeated and intentional language behavior directed at identifiable

									targets; emotional harm is discussed conceptually rather than measured directly from victims.
Xu and Trzaskawka (2021)	Journal article; interdisciplinary case analysis	Cross-national cases involving China, Nigeria, France and Poland	Selected authentic cyberbullying cases	Interdisciplinary legal, linguistic and case-based analysis	Cyberbullying overlapped with hate speech, trolling and stalking but was distinguished through targeting, intention, repetition and harm. The analysis highlighted conceptual ambiguity across disciplinary definitions.	Ambiguous definitions complicated identification, legislation and platform intervention. The study emphasized the need to assess communicative behaviour together with repetition and the target's capacity to experience harm.	Cyberbullying, hate speech, trolling, stalking, cyber harassment, deindividuation	Specific individuals or groups (e.g., hunters, foresters, or specific case victims across different countries)	Evidence: Amplification discussed through deindividuation in group chats, retweets, and 'likes' that perpetuate a victim's bullying experience online. Rationale: Fits because it isolates cyberbullying as a distinct construct defined by the intersection of targeting,

									intent, repetition, and a victim's capacity to experience harm.
Li and Peng (2024)	Journal article	Chinese social media; no single platform specified	Questionnaire responses from 705 participants and selected textual examples	Content analysis and questionnaire analysis	The study distinguished explicit textual cues, including offensive expressions, pronouns and negation, from implicit sentimental cues. Negative affect was dominated by anger, followed by anxiety and sadness.	Differences were reported in users' recognition of direct and implicit cues. The findings supported combining linguistic and affective information when examining perceptions of cyberbullying.	Cyberbullying (victimization and perpetration), flaming, online harassment, roasting, 'imbalanced relation'	Individual/group targets across selected cases (e.g., Chinese Women's Volleyball Team, actor, female VOGUE model, pediatrician)	Evidence: Users prefer repetitively using strong offensive words (e.g., 'whore', 'bitch', 'go die') with negative sentiments to achieve cyberbullying goals. Rationale: Fits because it redefines cyberbullying as an 'imbalanced relation' of repeated, aggressive language behavior utilizing digital

									deindividuation.
Zhong et al. (2022)	Journal article	Weibo, WeChat, QQ, TikTok and Toutiao	23,980 comments from five cyberbullying cases	Lexical analysis, sentiment analysis and semantic-network analysis	Swear words, second-person pronouns and emotional expressions were recurrent. Negative sentiment was dominant, while apparently positive expressions could communicate sarcasm. Semantic networks clustered around gender, wealth and social-identity conflicts.	Explicit and implicit aggression varied across cases and platforms. Collective commentary contributed to escalation, while moral positioning influenced whether users framed participation as aggression or justified criticism.	Cyberbullying (explicit/implicit), online moral failure, cyber aggression, deindividuation, explicit and implicit bullying	Individual targets of 5 cases across social domains (Education: Tsinghua student; Entertainment: actress; Society: COVID-19 trip; Finance: Mr. X; Sports: Miss M)	Evidence: Multi-user 'collective commentary' was associated with escalation across the five cases, while retweets, 'likes', tagging and hashtags supported distributed repetition and wider circulation of negative evaluations. Rationale: Fits because the study documents target-specific cases in which collective

									participation functions as a form of repetition/amplification. Victim-reported trauma or psychological outcomes were not directly measured in the linguistic and network-oriented analysis.
Lu et al. (2022)	Journal article	Sina Weibo	53,526 posts collected over one month	Sentiment analysis and latent Dirichlet allocation topic modelling	Negative emotional language was prominent, with anger occurring more frequently than anxiety and sadness. The dataset also contained	Weibo supported competing forms of audience behaviour, including condemnation, emotional reaction, support for	Cyberbullying, negative/angry public attitude, cyber manhunt, celebrity worship	Specific celebrities/stars (such as actor Zhan Xiao and his fans) and victims of public events	Evidence: Large-scale collective participation (53,526 posts) and 'cyber manhunt' (人肉搜索) were accompanied by accumulating public

	supportive discourse and calls for intervention.	targets and demands for stronger control. The evidence showed that audience responses were not uniformly aggressive.		comments, rumors and angry opinions around identifiable public-event targets. Rationale: Fits because the source documents repeated, hostile and collectively distributed communicatio n; the sentiment analysis measures public discourse rather than victims' emotional distress directly.

Zhang (2020)	Journal article	WeChat subscription accounts	Twenty-six WeChat texts comprising 18,722 tokens and 4,266 word types	Corpus-based lexical, semantic-prosody and pragmatic analysis	Negative lexical clusters, direct-address forms and cumulative semantic prosody generated hostility. The illocutionary force of apparently evaluative discourse developed from flaming into incitement to offline harm.	The extended format of WeChat subscription content allowed negative framing to accumulate across longer discourse. Repeated associations encouraged collective hostility and potential offline action.	Cyberbullying, flaming, internet incitement, language crime, 'dog fans' (cyberspeak)	Group targets: 'Dog fans' (dog lovers) and front-line police/officials in dog-related controversies	Evidence: The extended format of WeChat subscription content allowed negative framing to accumulate across longer discourse, and repeated associations on public social media multiplied collective hostility. Rationale: Fits as indirect, covert cyberbullying that repeatedly targets a group, creating an offline risk of incitement.
Chen et al. (2024)	Journal article	Chinese social media	Expanded COLDATASET	XLNet contextual	Contextual and sequential representations	The findings indicated that context-	Cyberbullying, hate speech, explicit/implicit	Specific individuals from 10 real	Evidence: Incident-based comments

	data, including incident-based comments and Douban reviews	totaling 38,991 texts (28,991 training, 5,000 validation and 5,000 test). The final total includes approximately 1,660 offensive remarks collected from ten real cyberbullying incidents during dataset expansion, together with Douban one-star reviews.	embeddings combined with Deep Bi-LSTM; comparison with SVM, GRU and BERT baselines	captured dependencies associated with implicit offensive language. The hybrid model achieved an F1 score of 90.43%.	sensitive modelling was more effective than isolated lexical features for identifying implicit aggressive communication. Model performance was treated as supplementary evidence.	bullying, 'keyboard warriors', social distrust, cyberstalking	cyberbullying incidents (e.g., Zheng Linghua, Liu Xuezhou, Wuhan Sugar Water Grandpa)	included implicit hostile remarks such as mockery, innuendo and rhetorical questions directed at identifiable individuals; the expanded dataset captures repeated forms of offensive communication across ten real cyberbullying incidents. Rationale: Fits because it provides target-directed, context-sensitive evidence of

									persistent or recurrent online aggression. The source describes serious victim consequences in the incident contexts, but the computational analysis did not directly measure victim mental health, experienced harm or power asymmetry.
**Chao et al. (2024)**	Journal Article (Heliyon)	PTT (Taiwan); pandemic-related hate speech regarding vaccines	16,967 comments collected within a 14-day window of significant domestic	Two-tier machine learning (ML), ChatGPT rephrasing, and LDA topic modeling.	Identified 30 pandemic-related terms; LLMs effectively reduce hostility by substituting derogatory terms with generalizations	Initial hate speech focused on bias, fear, and hostility; PTT's upvote/downvote mechanisms were omitted to focus purely on comment content.	Chinese language abuse, hate speech, offensive/derogatory language, cyberbullying	Specific social groups and entities (e.g., Wanhua district residents, legislators, flight crews, specific	Evidence: Across 16,967 comments collected during a 14-day event window, recurrent derogatory

	and policies.	pandemic events.	while preserving the original intent.		political parties/adherents, and the elderly)	labels and hostile expressions were directed at identifiable social groups and entities. The study analyzed comment content and did not directly measure reposting, interactional repetition or platform-level amplification. Rationale: Retained as adjacent evidence because it documents recurring, target-directed harmful language across a

									temporally concentrated public-event context; it is used primarily as linguistic evidence rather than as direct evidence of victim-level repetition.
**Jin and Tay (2023)**	Journal Article (Discourse Studies)	Sina Weibo; public blame directed at the Hubei Red Cross Foundation during the first COVID-19 wave.	14,540 comments containing 292,253 Chinese characters.	Discursive Psychology (DP) and rhetorical analysis.	Users employ "evidential blame" to frame hate speech as a legitimate punishment; the term "blackhearted" is a key rhetorical device used to challenge institutional norms.	Hateful comments function as a networked practice of blame; the platform allows ordinary users to engage in socio-political discussions in a decentralized manner.	Offensive and hateful comments, evidential blame, radical extremist speech, 'legitimate complainable/hateable', dehumanisation	Institution target: The Hubei Red Cross Foundation (HBRCF) and its officials	Evidence: Networked practice of collective blame where comments build on each other to form a 'joint construction of hate speech' (e.g., comments repeating and upgrading previous ones, implying nationwide consensus).

									Rationale: Fits as a collaborative digital practice that functions as a radical, offensive, and collective social movement of moral regulation.
**Liu et al. (2021)**	Journal Article (Asian Journal of Comm.)	Sina Weibo; online anti-African sentiments and disease-related misinformation.	102,560 initial posts; top 50 popular posts per keyword group (e.g., heiren, AIDS).	Automated scraping and Critical Discourse Analysis (CDA).	Anti-African discourse is built on three nodal points: "promiscuous" African men, "unchaste" Chinese women, and unidirectional virus transmission.	Racist sentiments are mixed with gendered anxiety; posts primarily originate from regions with high African immigrant populations like Beijing, Shanghai, and Guangdong.	Online racist discourse, cyber racism, othering, online hatred, 'three-non-people'	Group target: China-based Africans (and Chinese women who date them)	Evidence: Rapid spread and amplification of anti-African discourses on Weibo (collecting over 102,560 posts), where posts warning about 'dangerous black men' received tens of thousands of retweets and comments. Rationale: Fits as a cyber-racist hate

									speech campaign that acts as a container and catalyst for collective prejudice and othering.
**Lu et al. (2023)**	Conference Paper (ACL)	Zhihu and Tieba; fine-grained Chinese toxic language detection across sensitive topics.	12,011 comments (ToxiCN dataset) covering gender, race, region, and LGBTQ.	Hierarchical taxonomy (Monitor Toxic Frame) and TKE benchmarking.	Distinction between hate speech and general offensive language; documented 1,032 terms including implicit irony and metaphors (e.g., "fairy" used to attack women).	Zhihu users favor subtle expressions (implicit/reporting), while Tieba users employ more direct attacks (explicit/offensive).	Chinese toxic language, general offensive language, hate speech (sexism, racism, regional bias, anti-LGBTQ), implicit/explicit profanity	Diverse social groups (Sexist targets, regional targets e.g. Henan people, racial targets, LGBTQ community)	Evidence: The ToxiCN dataset contains recurrent explicit and implicit target-directed toxic expressions across gender, race, region and LGBTQ categories, including culturally loaded variants such as metaphorical labels. The

				study does not directly measure repeated targeting of the same individual or platform amplification. Rationale: Retained as adjacent linguistic evidence because it distinguishes hate speech from general offensive language and documents recurring group-directed hostility; it is not treated as direct evidence of victim-level persistence.

**Xia and Wang (2022)**	Journal Article (Discourse, Context & Media)	Baidu Post Bar; use of the creative trolling label "*Gangjing*".	40 interactional threads selected from 6,534 results.	Conversation Analysis (CA) and pragmatic analysis.	"Gangjing" serves as a negative stance marker used to show disagreement, delegitimize others as irrational, or invite people for jocular abuse.	Trolling labels trigger responses like resisting, mocking, or attacking; Baidu's "floor" structure makes sequential interactional analysis easier.	Trolling, trolling label 'Gangjing' (杠精), communicative action, deindividuation, jocular abuse, relational acts	Individual targets within Baidu forums (e.g., users in game communities, tech forums, or those labeled 'Gangjing' in arguments)	Evidence: Labeling someone as 'Gangjing' is an interactional, repetitive practice (over 700k posts within a week). It triggers defensive/offensive loops of mocking, attacking, and escalating verbal aggression. Rationale: Fits as a metapragmatic label and trolling practice where participants perform deviant behaviors to disrupt or cause conflict for amusement.

**Zhang et al. (2024)**	Journal Article (IEEE Access)	Multi-platform (Weibo, Zhihu, WeChat, Xiaohongshu); implicit hate speech detection.	19,089 texts (MCIHD dataset) across nine domains including race, gender, and religion.	Domain-Enhanced Prompt Learning (DePL) and few-shot learning.	Implicit hate speech is characterized by "cold language" that is indirect, abstract, and ambiguous; domain-specific proprietary rhetoric varies by social category.	Prompt-based models exhibit significant advantages in low-data (few-shot) scenarios; multi-domain specificity makes hate speech harder to detect.	Chinese implicit hate speech, domain-specific proprietary rhetoric, explicit hate, 'cold language'	Specific individuals or groups across nine domains (including race, gender, religion, region, education, and occupation)	Evidence: The 19,089-text MCIHD dataset documents explicit and implicit hate speech directed at identifiable social categories across nine domains, including indirect, abstract and ambiguous 'cold language'. The computational design does not directly measure repeated targeting of the same person or platform-level collective

									amplification. Rationale: Retained as adjacent linguistic evidence because it provides target-directed harmful rhetoric across recurrent domain categories and is used to inform implicit-language mechanisms rather than to establish victim-level repetition.
**Fang et al. (2024)**	Journal Article (PLOS ONE)	Sina Weibo; diffusion of feminist discourses	366,602 network communication nodes within a	Complex network modeling and propagation dynamics.	Discursive content is categorized into information, opinion, and appeasement; opinion-based posts have higher netizen	Information diffusion follows a scale-free characteristic where a "key minority" of nodes (e.g., authoritative	Feminist discourse, hate speech, online gender-related disputes,	Group/Case targets: Feminist groups/women, victims of the 2022	Evidence: Measured through complex network modeling of

		following the 2022 Tangshan restauran t attack.	one-week period.		participation and lower average information decay.	media) significantly impacts spread.	misogyny, post-feminism	Tangshan restaurant assault, and public safety entities	366,602 user forwards. Propagation of opinion-based posts showed extremely high netizen participation, low information decay, and widespread dissemination driven by a 'key minority' of nodes. Rationale: Fits as it examines online gender disputes, victim-blaming, and misogynistic hate speech that are collectively amplified.
**Meng et al. (2026)**	Journal Article (Scientifi	Douyin, Bilibili, and Sina Weibo;	1,270 participant s across three	Scenario-based experimenta l design and	Examined individual behaviors to reduce participation in cyberbullying as a	IP location display suppresses cyberbullying by increasing	Cyberbullying behavior, deindividuation , impression	Individual targets of comments across major	Evidence: Three scenario-based

c Reports)	impact of mandatory IP location disclosure.	stepwise experimental studies.	multiple regression analysis.	defensive impression construction to avoid image damage.	impression management motivation and privacy concerns; this effect is stronger for those with high group identity	management motivation, moral disengagement, privacy concerns	social media platforms (Douyin, Bilibili, Sina Weibo)	experiments (1,270 participants in total) tested how mandatory IP-location disclosure was associated with cyberbullying participation through impression-management motivation and privacy concerns. Rationale: Fits because it directly examines behavioral mechanisms associated with committing or refraining from cyberbullying

across Douyin, Bilibili and Sina Weibo. This source provides experimental evidence about participation conditions rather than direct measurement of victim harm or collective amplification.

Note: Publication-type coding yields 16 journal articles and three full-length conference papers, with no book chapters. Xu is standardized to the first-online year 2020 for consistency with the revised manuscript. For Chen et al. (2024), the approximately 1,660 incident-based offensive remarks are included within the final expanded COLDATASET total of 38,991 texts, not additional to that total. Meng et al. is standardized to the final bibliographic year 2026; the article was first published online on 16 December 2025 and remained eligible under the 2015–2025 first-online publication rule. Where included sources did not directly measure victim-reported harm, power asymmetry, repeated targeting of the same individual, or platform-level amplification, the corresponding cells distinguish source-reported evidence from review-level interpretation.
