# Supplementary material for: Linguistic and Behavioral Mechanisms of Cyberbullying Among Chinese Social Media Users: A Scoping Review
Source: Behav Sci (Basel). 2026 Sep 11;16(9):1627. doi: 10.3390/bs16091627 (PMC13603809; doi:10.3390/bs16091627)
Supplement: Supplementary file 1 [file behavsci-16-01627-s001.zip › File S3.New PRISMA_2020_flow_diagram.pdf]

PRISMA Flow Diagram for Scoping Reviews

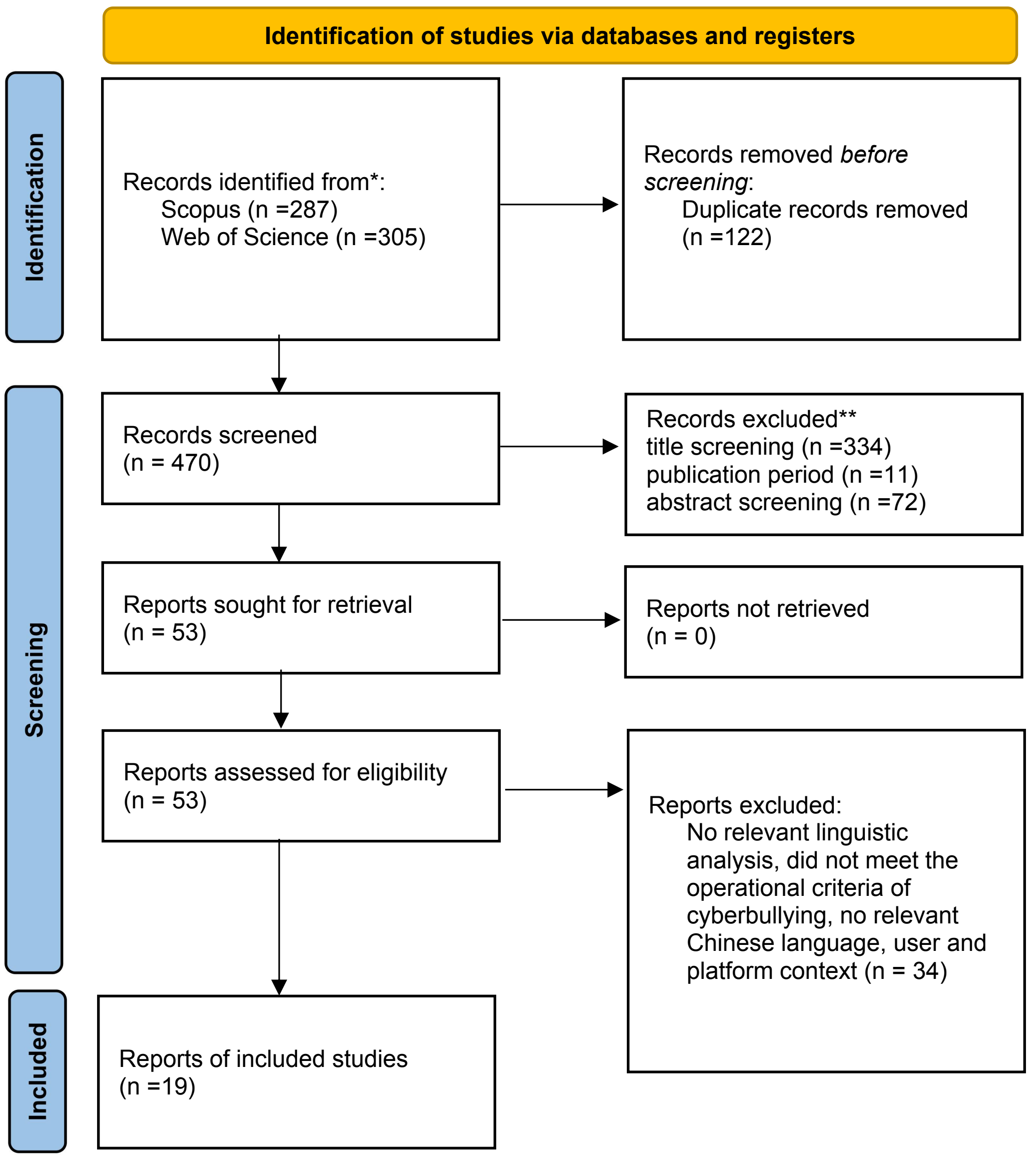

Figure 1. PRISMA flow diagram of study selection process.

Note: Flow diagram adapted from Tricco et al. (2018), PRISMA Extension for Scoping Reviews (PRISMA-ScR)
