# Supplementary material for: Linguistic and Behavioral Mechanisms of Cyberbullying Among Chinese Social Media Users: A Scoping Review
Source: Behav Sci (Basel). 2026 Sep 11;16(9):1627. doi: 10.3390/bs16091627 (PMC13603809; doi:10.3390/bs16091627)
Supplement: Supplementary file 1 [file behavsci-16-01627-s001.zip › File S4.Updated_Scoping_Review_Protocol.pdf]

Updated Scoping Review Protocol: Linguistic and Behavioral Mechanisms of Cyberbullying Among Chinese Social Media Users

Aligned with the revised Behavioral Sciences manuscript (2026)

**Protocol status**	Updated protocol aligned with the revised manuscript; methodological clarifications incorporated after peer review.
**OSF registration**	DOI:10.17605/OSF.IO/Q9M5A (registered in 2026).
**Review type**	Scoping review.
**Databases**	Scopus and Web of Science.
**Search date**	15 March 2026.
**Eligibility window**	English-language publications first released online between 2015 and 2025.
**Reporting guideline**	PRISMA-ScR (Tricco et al., 2018) as the sole reporting checklist.

Protocol note. This updated protocol records the methodological procedures and interpretive safeguards reflected in the revised manuscript. It clarifies the English-language/database scope, the handling of adjacent constructs, the treatment of publication-level recurrence and author concentration, and the distinction between directly measured victim outcomes and analyst-inferred communicative harm. These clarifications do not redefine the core review question or search corpus.

1. Review Design and Reporting Framework

This study employed a scoping review design to map and synthesize the available evidence on the linguistic and behavioral mechanisms of cyberbullying involving Chinese users on social media. A scoping review was selected because the evidence base is heterogeneous in terminology, disciplinary orientation, theoretical framework, research design, analytical method and platform coverage. The purpose was therefore to characterize the extent and nature of the available evidence, clarify conceptual boundaries, identify recurrent mechanisms and map research gaps rather than estimate prevalence, evaluate intervention effectiveness or generate pooled effect sizes.

The review was conducted using the methodological framework developed by Arksey and O'Malley (2005) and subsequently refined by Levac et al. (2010). This framework informed formulation of the review question, identification of relevant sources, study selection, data charting and synthesis. The Population-Concept-Context (PCC) framework was used to structure the scope, eligibility criteria and search concepts (Peters et al., 2020).

Reporting of the completed review was guided by the Preferred Reporting Items for Systematic Reviews and Meta-Analyses Extension for Scoping Reviews (PRISMA-ScR; Tricco et al., 2018). PRISMA-ScR is used as the sole reporting checklist for the scoping review. The identification, screening, eligibility assessment and final inclusion of sources are documented in a source-selection flow diagram, and the optional critical-appraisal items are treated as not applicable because no formal study-level critical appraisal was undertaken.

2. Review Aim and Research Questions

The review aims to map how cyberbullying involving Chinese users is linguistically enacted and behaviorally reinforced across social media environments, while explicitly delimiting the evidence to English-language studies indexed in Scopus and Web of Science. The four research questions are:

RQ1. What are the main characteristics, methodological approaches and social media contexts of indexed English-language research on cyberbullying involving Chinese users?

RQ2. What recurrent linguistic and behavioral mechanisms of cyberbullying are identified across the included sources?

RQ3. How do interactional context and platform affordances relate to the interpretation, reinforcement and amplification of cyberbullying in the reviewed literature?

RQ4. What methodological, platform, language and evidence-base biases or gaps limit interpretation and transferability beyond the indexed English-language evidence base?

3. Population-Concept-Context Framework

Population. The population comprises users who produce, receive or are represented in Chinese-language social media communication. This includes Chinese-speaking users residing within and outside mainland China. Eligibility is determined primarily by the linguistic and communicative context examined rather than by assumptions concerning nationality or geographical location.

Concept. The concept encompasses linguistic and behavioral mechanisms through which cyberbullying is enacted, reinforced, interpreted or legitimized. Linguistic mechanisms include lexical and lexico-semantic choices, grammatical constructions, metaphors, pragmatic strategies and discursive processes through which harmful evaluation acquires contextual meaning (Culpeper, 2011; Xu, 2020). Behavioral mechanisms include direct targeting, repeated negative evaluation, participant alignment, norm enforcement, moderation evasion and collective amplification.

Context. The context comprises interactions occurring on Chinese and international social media platforms that contain relevant Chinese-language communication, including Weibo, WeChat, Douyin or TikTok, Zhihu, Bilibili and other comparable social media environments.

4. Operational Definition and Adjacent Constructs

For study selection, cyberbullying is operationally defined as target-directed online communication that causes, intends to cause or has the capacity to cause social, emotional or reputational harm. Drawing on established conceptualizations, eligible communication must involve an identifiable individual or group target, harmful linguistic or discursive conduct, repetition, persistence or collective amplification, and an asymmetrical capacity to harm or marginalize the target (Kowalski et al., 2014; Xu & Trzaskawka, 2021).

Repetition is not restricted to recurring messages from one perpetrator. In networked social media environments it may also arise through reposting, tagging, coordinated commentary, repeated labelling or cumulative participation by multiple users. Such distributed recurrence is considered relevant when public visibility and audience participation permit isolated expressions to become part of a collective and persistent attack (Xu, 2020; Xu & Trzaskawka, 2021).

Studies using adjacent concepts such as online harassment, hate speech, toxic language, trolling, flaming or public shaming are considered only when the communication examined satisfies the operational criteria above. Terminological similarity or the presence of generally offensive language is insufficient for inclusion. This decision rule distinguishes cyberbullying from isolated insults, non-targeted toxic language, general ideological disagreement and harmful communication lacking evidence of repetition, collective amplification or asymmetrical harm (Hardaker, 2010; Ortiz, 2020; Xu & Trzaskawka, 2021).

Evidence-status safeguard. During charting and synthesis, directly measured or participant-reported victim outcomes are distinguished from analyst-inferred communicative harm and power asymmetry. When a source infers harm or asymmetry from textual patterns, audience size, public visibility, repeated targeting or collective participation, the synthesis will describe these as indicators or inferred communicative conditions rather than as established experienced harm. Claims of experienced harm will be reserved for studies that directly measure or report victim interpretations or outcomes.

5. Information Sources and Search Strategy

Scopus and Web of Science were selected deliberately to define a transparent and reproducible corpus of internationally indexed, peer-reviewed scholarship across linguistics, communication, behavioral sciences, social sciences and computational research. Their field-specific search functions, Boolean operators and filtering facilities support precise database-specific retrieval. This database choice is not intended to provide exhaustive coverage of scholarship on Chinese-language cyberbullying. For a linguistically focused review, restricting the evidence base to internationally indexed English-language sources carries a substantive validity cost because locally published Chinese-language research may contain more granular analyses of platform-specific slang, homophones, metaphors, pragmatic meanings and culturally situated interaction. Scopus and Web of Science are therefore treated as defining a bounded indexed evidence slice rather than the entire field.

The search strategy contains three conceptual blocks: (1) online aggression, (2) Chinese linguistic or platform contexts, and (3) linguistic or discourse analysis. Adjacent terms such as hate speech, toxic language, trolling and flaming are included to improve search sensitivity, but their presence in a title, abstract or keywords does not itself establish eligibility. Studies retrieved through broader terms remain subject to the operational cyberbullying criteria during screening.

Table 1. Database-specific search strings.

Database	Complete search string
**Scopus**	TITLE-ABS-KEY ((cyberbully* OR "online aggression" OR "online harassment" OR "digital bullying" OR "internet bullying" OR "abusive language" OR "toxic language" OR "hate speech" OR "online hate speech" OR trolling OR flaming OR "public shaming") AND (Chinese OR China OR Mandarin OR Cantonese OR "Chinese language" OR "Chinese-language" OR Weibo OR WeChat OR Douyin OR TikTok OR Zhihu OR Bilibili) AND (language OR linguist* OR lexical* OR semantic* OR pragmatic* OR discourse OR metaphor* OR impoliteness OR "speech act*" OR communication OR interaction))
**Web of Science**	TS=((cyberbully* OR "online aggression" OR "online harassment" OR "digital bullying" OR "internet bullying" OR "abusive language" OR "toxic language" OR "hate speech" OR "online hate speech" OR trolling OR flaming OR "public shaming") AND (Chinese OR China OR Mandarin OR Cantonese OR "Chinese language" OR "Chinese-language" OR Weibo OR WeChat OR Douyin OR TikTok OR Zhihu OR Bilibili) AND (language OR linguist* OR lexical* OR semantic* OR pragmatic* OR discourse OR metaphor* OR impoliteness OR "speech act*" OR communication OR interaction))

The database searches were conducted on 15 March 2026. All queries were saved and record counts were documented before deduplication. Publication-year and language criteria were applied during screening rather than embedded in the database queries. Eligible sources were limited to English-language publications within the 2015-2025 eligibility window. For publications first released online within that window but assigned to a later journal volume, the first online publication date was used to determine eligibility. Chinese-language databases and locally published scholarship were outside the registered search scope and are treated as a substantive evidence limitation rather than a technical retrieval issue.

6. Eligibility Criteria

Eligibility is assessed at title/abstract and full-text stages using the PCC framework and the operational definition above. The criteria are summarized in Table 2.

Table 2. Inclusion and exclusion criteria.

Dimension	Include	Exclude
**Topic**	Cyberbullying or substantively equivalent target-directed online aggression.	Offline bullying; general social media behavior without harmful target-directed communication.

Dimension	Include	Exclude
**Population/context**	Chinese-language users or Chinese-language communication on social media.	Non-Chinese contexts without a relevant Chinese-language or Chinese-user component.
**Linguistic/behavioral focus**	Meaningful analysis of lexical, lexico-semantic, grammatical, pragmatic, interactional, discursive, behavioral or platform-mediated mechanisms.	Prevalence, demographics or psychological outcomes only, without communicative analysis.
**Adjacent constructs**	Harassment, hate speech, toxic language, trolling, flaming or public shaming only when identifiable target, harmful conduct, repetition/persistence/collective amplification and asymmetrical capacity to harm are evidenced.	General offensive/toxic language without the substantive operational conditions for cyberbullying.
**Study design**	Original empirical research using qualitative, quantitative or mixed methods.	Reviews, editorials, book reviews, commentaries or non-research publications.
**Publication type**	Peer-reviewed journal articles, full-length conference papers and scholarly book chapters reporting original empirical research.	Dissertations, theses, conference abstracts and publications with insufficient methodological information.
**Technical studies**	Computational studies with interpretable linguistic, contextual, sequential or interactional findings.	Software architecture, model optimization or classification performance only.
**Language/time**	English-language publications first released online in 2015-2025.	Non-English publications; publications outside the eligibility window.

7. Study Selection and Screening

All records retrieved from Scopus and Web of Science are exported to EndNote for reference management and deduplication. Potential duplicates are identified using title, authorship, publication year and digital object identifier, followed by manual checking before removal.

Study selection proceeds in two sequential stages: title/abstract screening followed by full-text eligibility assessment. Two reviewers participate in screening. When title or abstract information is insufficient to determine eligibility, the record is retained for full-text assessment to minimize premature exclusion. Differences in eligibility judgements are discussed until consensus is reached.

During abstract screening, records are excluded when they focus exclusively on offline bullying, non-Chinese contexts, cybersecurity infrastructure, general social media behavior, psychological outcomes or prevalence without communicative analysis. General sentiment, offensive-language detection and toxicity studies are excluded when they do not establish an identifiable relationship with cyberbullying or substantively equivalent target-directed online aggression. At full text, each source must meet the operational criteria and provide interpretable linguistic or behavioral evidence.

8. Data Charting

Data are charted using a standardized extraction form developed from the review questions and PCC framework. The form records authorship, publication year, publication type, geographical and linguistic context, social media platform, dataset or sample, analytical method, theoretical framework and terminology used to describe harmful behavior.

Linguistic and lexico-semantic fields include explicit offensive expressions, evaluative labels, metaphors, homophones, misspellings and context-dependent meanings. Pragmatic and discursive fields include implicature, presupposition, sarcasm, semantic prosody, identity construction, moral positioning and other forms of indirect aggression. Behavioral and platform-related fields include direct targeting, repeated evaluation, participant alignment, moderation evasion, collective participation, reposting and other forms of platform-mediated amplification.

The charting form also records whether and how each source establishes: (a) an identifiable target; (b) harmful communicative conduct; (c) repetition, persistence or collective amplification; and (d) asymmetrical capacity to harm. A separate evidence-status field distinguishes directly measured or participant-reported harm/power asymmetry from analyst-inferred indicators. Authorship is retained as a charting field so that publication-level recurrence from the same author cluster can be identified during synthesis.

For computational studies, only interpretable findings concerning linguistic, contextual, sequential or interactional features are incorporated into the principal synthesis. Classification accuracy, F1 scores and model comparisons are treated as supplementary evidence and are not interpreted as direct evidence of linguistic or behavioral mechanisms.

9. Critical Appraisal and Risk of Bias

No formal methodological quality, critical appraisal or study-level risk-of-bias assessment is undertaken because the purpose of this scoping review is to map the nature, characteristics and extent of the available evidence rather than determine an aggregate intervention effect. The optional PRISMA-ScR critical-appraisal items are therefore reported as Not applicable. Nevertheless, study design, dataset size, platform coverage, analytical method and transparency of data collection are charted and considered when interpreting the findings. The absence of formal critical appraisal is acknowledged as a limitation of the review.

10. Evidence Synthesis

Analysis proceeds in two stages. First, descriptive mapping summarizes publication years, publication types, platforms, data sources, methodological approaches, theoretical orientations and analytical traditions. Frequencies are reported only for characteristics that are sufficiently comparable across sources and are not interpreted as statistically generalizable estimates.

Second, thematic synthesis is conducted using an initial analytical framework derived from the review questions. The framework comprises lexical and lexico-semantic realization, pragmatic meaning, discursive identity construction, behavioral reinforcement and platform affordances. Findings within these domains are coded and compared to identify recurrent patterns, contextual variation, divergent evidence and research gaps. Related codes are consolidated into higher-order themes describing how cyberbullying is linguistically enacted, behaviorally reinforced, socially legitimized and amplified through platform-mediated interaction within the indexed evidence base.

The synthesis is organized around patterns recurring across publications rather than sequential summaries of individual studies. For each theme, contributing sources, areas of thematic consistency, platform- or method-specific differences and evidential limitations are recorded. Author concentration is treated as an interpretive sensitivity: recurrence across multiple publications linked to the same author cluster is not treated as equivalent to independent replication across unrelated research teams. Patterns recurring across distinct author groups and analytical traditions are given greater interpretive weight as evidence of thematic consistency.

The resulting framework is explicitly provisional. It represents relationships documented within the included English-language Scopus/WoS evidence slice and is not presented as a causal, universal or field-comprehensive model. Relationships between linguistic realization and contextual/pragmatic interpretation, and between platform conditions and patterns of visibility or discourse circulation, may be synthesized where recurrently documented. Longer sequences linking pragmatic interpretation, participant alignment, collective amplification and experienced harm are treated as integrative propositions for future research unless directly tested.

11. Alignment of Data with the Research Questions

- RQ1 will be addressed through descriptive mapping of publication characteristics, study design, theoretical orientation, analytical method, platform and data source.

- RQ2 will be addressed through charted lexical, pragmatic, discursive and behavioral mechanisms and thematic synthesis of recurrent patterns.
- RQ3 will be addressed through charted interactional context, participant response, platform affordances, visibility, repetition and circulation.
- RQ4 will be addressed through comparison of methodological heterogeneity, platform coverage, language/database scope, victim-centered evidence, evidential status of harm/power asymmetry, source concentration and analytical transparency.

12. Evidence-Boundary and Interpretation Safeguards

The review is deliberately bounded to English-language publications indexed in Scopus and Web of Science. Accordingly, absence or low frequency of a linguistic or pragmatic pattern in the review will not be interpreted as evidence of its absence from Chinese-language scholarship. The synthesis and any framework developed from it will be described as deriving from this indexed English-language evidence slice, not from the field as a whole. Transferability to Chinese-language scholarship requires direct evaluation using Chinese-language databases and locally grounded studies.

The review will also distinguish publication-level recurrence from independent replication, and inferred communicative harm from directly measured victim outcomes. These safeguards are intended to prevent overstatement of thematic convergence, causal relationships, harm and generalizability.

13. Transparency, Registration and Data Availability

The review protocol was registered on Open Science Framework in 2026 (https://osf.io/q9m5a). The revised reporting package uses PRISMA-ScR consistently and includes the full database search strings, eligibility criteria, screening procedures, data-charting categories and approach to synthesis. Supporting data and materials for the review are available in Zenodo at https://doi.org/10.5281/zenodo.21628741, with additional review materials available through the OSF registration.

References

Arksey, H., & O'Malley, L. (2005). Scoping studies: Towards a methodological framework. International Journal of Social Research Methodology, 8(1), 19-32.

Culpeper, J. (2011). Impoliteness: Using language to cause offence. Cambridge University Press.

Hardaker, C. (2010). Trolling in asynchronous computer-mediated communication: From user discussions to academic definitions. Journal of Politeness Research, 6(2), 215-242.

Kowalski, R. M., Giumetti, G. W., Schroeder, A. N., & Lattanner, M. R. (2014). Bullying in the digital age: A critical review and meta-analysis of cyberbullying research among youth. Psychological Bulletin, 140(4), 1073-1137.

Levac, D., Colquhoun, H., & O'Brien, K. K. (2010). Scoping studies: Advancing the methodology. Implementation Science, 5(1), 69.

Ortiz, S. M. (2020). Trolling as a collective form of harassment: An inductive study of how online users understand trolling. Social Media + Society, 6(2), 2056305120928512.

Peters, M. D. J., Marnie, C., Tricco, A. C., Pollock, D., Munn, Z., Alexander, L., McInerney, P., Godfrey, C. M., & Khalil, H. (2020). Updated methodological guidance for the conduct of scoping reviews. JBI Evidence Synthesis, 18(10), 2119-2126.

Tricco, A. C., Lillie, E., Zarin, W., O'Brien, K. K., Colquhoun, H., Levac, D., et al. (2018). PRISMA extension for scoping reviews (PRISMA-ScR): Checklist and explanation. Annals of Internal Medicine, 169(7), 467-473.

Xu, Y. (2020). The invisible aggressive fist: Features of cyberbullying language in China. International Journal for the Semiotics of Law, 34, 1041-1064. https://doi.org/10.1007/s11196-020-09746-1

Xu, S., & Trzaskawka, P. (2021). Towards descriptive adequacy of cyberbullying: Interdisciplinary studies on features, cases and legislative concerns of cyberbullying. International Journal for the Semiotics of Law, 34(2), 533-552.
